# Oxylipin profile in saliva from patients with cystic fibrosis reveals a balance between pro-resolving and pro-inflammatory molecules

**DOI:** 10.1038/s41598-022-09618-7

**Published:** 2022-04-07

**Authors:** Vincenzo Carnovale, Alice Castaldo, Alessandro Di Minno, Monica Gelzo, Paola Iacotucci, Anna Illiano, Gabriella Pinto, Giuseppe Castaldo, Angela Amoresano

**Affiliations:** 1 Centro Di Riferimento Regionale Fibrosi Cistica, Naples, Italy; 2 Dipartimento Di Scienze Mediche Traslazionali, Università Di Napoli Federico II, Naples, Italy; 3 Dipartimento Di Farmacia, Università Di Napoli Federico II, Naples, Italy; 4 CEINGE-Biotecnologie Avanzate, Scarl, Naples, Italy; 5 Dipartimento Di Medicina Molecolare E Biotecnologie Mediche, Università Di Napoli Federico II, Naples, Italy; 6 Dipartimento Di Scienze Chimiche, Università Di Napoli Federico II, Naples, Italy; 7 Consorzio Interuniversitario “Istituto Nazionale Nazionale Biostrutture E Biosistemi (INBB)”, Rome, Italy

**Keywords:** Metabolomics, Biomarkers, Translational research

## Abstract

Oxylipins are signaling molecules originated by fatty acids that modulate vascular and bronchial tone, bronchial secretion, cytokine production and immune cell activity. The unbalanced production of pro-inflammatory and pro-resolving (i.e., anti-inflammatory) oxylipins has a relevant role in the pathogenesis of pulmonary inflammation like in cystic fibrosis (CF). We analyzed by LC-MRM/MS 65 oxylipins and 4 fatty acids in resting saliva from 69 patients with CF and 50 healthy subjects (controls). The salivary levels of 48/65 oxylipins were significantly different between CF patients and controls. Among these, EpETE, DHET, 6ketoPGE1 and HDHA were significantly higher in saliva from CF patients than in controls. All these molecules display anti-inflammatory effects, i.e., releasing of bronchial and vascular tone, modulation of cytokine release. While 20-hydroxyPGF2A, PGB2, EpDPE, 9 K-12-ELA, bicyclo-PGE2, oleic acid, LTC4, linoleic acid, 15oxoEDE, 20 hydroxyPGE2 and DHK-PGD2/PGE2 (mostly associated to pro-inflammatory effects) resulted significantly lower in CF patients than in controls. Our data suggest that the salivary oxylipins profile in CF patients is addressed toward a global anti-inflammatory effect. Although these findings need be confirmed on larger populations in prospective studies, they will contribute to better understand the pathogenesis of CF chronic inflammation and to drive targeted therapies based on the modulation of oxylipins synthesis and degradation.

## Introduction

The main research interest in the field of lipids concerns their metabolic activity and their role as structural and functional constituents of biological membranes. However, these functions represent only the tip of the iceberg of a multitude of activities related to the modulation of processes and pathways that include inflammation and immune response^1^. In particular, oxylipins are a group of signaling molecules mainly originated by fatty acids that display multisystemic regulatory activities including the modulation of vascular and bronchial tone, bronchial secretion, the regulation of cytokine production and release, and the modulation of immune cell activities. The unbalanced production and activity of pro-inflammatory and anti-inflammatory^2^ molecules have a relevant role in the pathogenesis of pulmonary inflammation^3^. The development of spectroscopic analysis technologies^4^ has given a significant boost to the study of targeted metabolomics, allowing to analyze large panels of lipid mediators^3^ also in emerging pulmonary diseases like Sars-CoV-2 infection^5^. However, most studies related the lipidomic profile to chronic pulmonary diseases like chronic obstructive pulmonary disease, allergic diseases, and cystic fibrosis (CF) helping to reveal the complex crosstalk among these molecules and inflammatory and immune pathways^3,6^.

Cystic fibrosis is a frequent, severe and multisystemic disease. Its hallmark is chronic inflammation that involves most tissue and organs among which lung with the impairment of airway secretions that compromise mucociliary clearance. The release of inflammatory mediators acting as chemoattractant and the chronic colonization by opportunistic strains, particularly in genetically predisposed subjects^7^, create a vicious circle of chronic airway inflammation that causes destruction of lung tissue leading to bronchiectasis and consequent respiratory insufficiency that represents the most frequent cause of death in patients with CF^8,9^.

A series of studies evaluated the lipidomic profile in airway secretions and in sputum from patients with CF revealing a network of pro-inflammatory and anti-inflammatory molecules^6,10^ usually unbalanced toward pro-inflammatory modulators^11^. These studies aid to understand the pathogenesis of chronic inflammation in patients with CF but may also translationally contribute to reveal sensitive biomarkers for the prognostic evaluation of patients with CF. Furthermore, a better knowledge of the oxylipins pathway in patients with CF helps to handling and monitoring therapeutic approaches that may target the different steps of the biosynthesis and degradation of these molecules^12–15^, or more, may be based on the direct use of these molecules^16,17^.

However, bronchoalveolar lavage (BAL) fluid as like as sputum are difficult to collect, particularly in CF children, as it is difficult to obtain control samples from healthy subjects. Furthermore, sputum, BAL and exhaled breath may be contaminated by saliva^18,19^. For this reason, our group approached to analyze resting saliva that is easily and non-invasively collected also from small children with CF, demonstrating that salivary cytokine levels relate with the severity of pulmonary and sinonasal disease^20^. Later, we demonstrated that unsaturated/saturated non esterified fatty acid (NEFA) ratio in saliva of patients with CF may represent a prognostic marker of lung disease^21^.

In the present study, we analyzed a large oxylipins profile and total fatty acids in resting saliva from a group of patients with CF and from a control group of healthy subjects relating the results to the severity of the pulmonary disease in patients with CF.

## Results

Supplementary Table 1 reports the comparison of the salivary levels of oxylipins (n = 65) and total fatty acids (n = 4) between patients with CF and healthy subjects (controls). This comparison showed that the salivary levels of 48/65 oxylipins were significantly different between CF patients and controls. The levels of unsaturated fatty acids, i.e., oleic and linoleic acids, were significantly lower in CF patients than in controls, while the saturated fatty acids (palmitic and stearic acids) levels did not show significant differences between the two groups.

In addition, we found significantly higher levels of serum C reactive protein (CRP) and salivary cytokines, i.e., interleukin (IL)-6, IL-8, tumor necrosis factor (TNF) α, in CF patients as compared to controls (Supplementary Table 2). The Spearman correlation analysis showed different significant associations between inflammation biomarkers and oxylipins in CF patients (Supplementary Table 3). Among the differentially expressed oxylipins, we observed that EpETE was positively correlated with IL-6 and HOTrE, PGE2/PGD2, LXA5, and LXA4 with IL-8, while other oxylipins were negatively associated with the inflammation biomarkers. In controls, we found only two significant correlations between IL-8 *versus* 7(R)-maresin-1 (p: 0.002, r_s_: 0.732) and resolvin D (p: 0.043; r_s_: −0.456).

Then, we performed PCA analysis using the concentrations values of all oxylipins and total fatty acids. PCA 2D score plot showed that CF patients clustered in a zone overlapping partially the control (CTR) zone (Supplementary Fig. 1-A). While the PCA synchronized 3D plot (Supplementary Fig. 1-B) showed that the first (PC1), the second (PC2) and the third (PC3) components separated CF patients from CTR.

**Figure 1:**
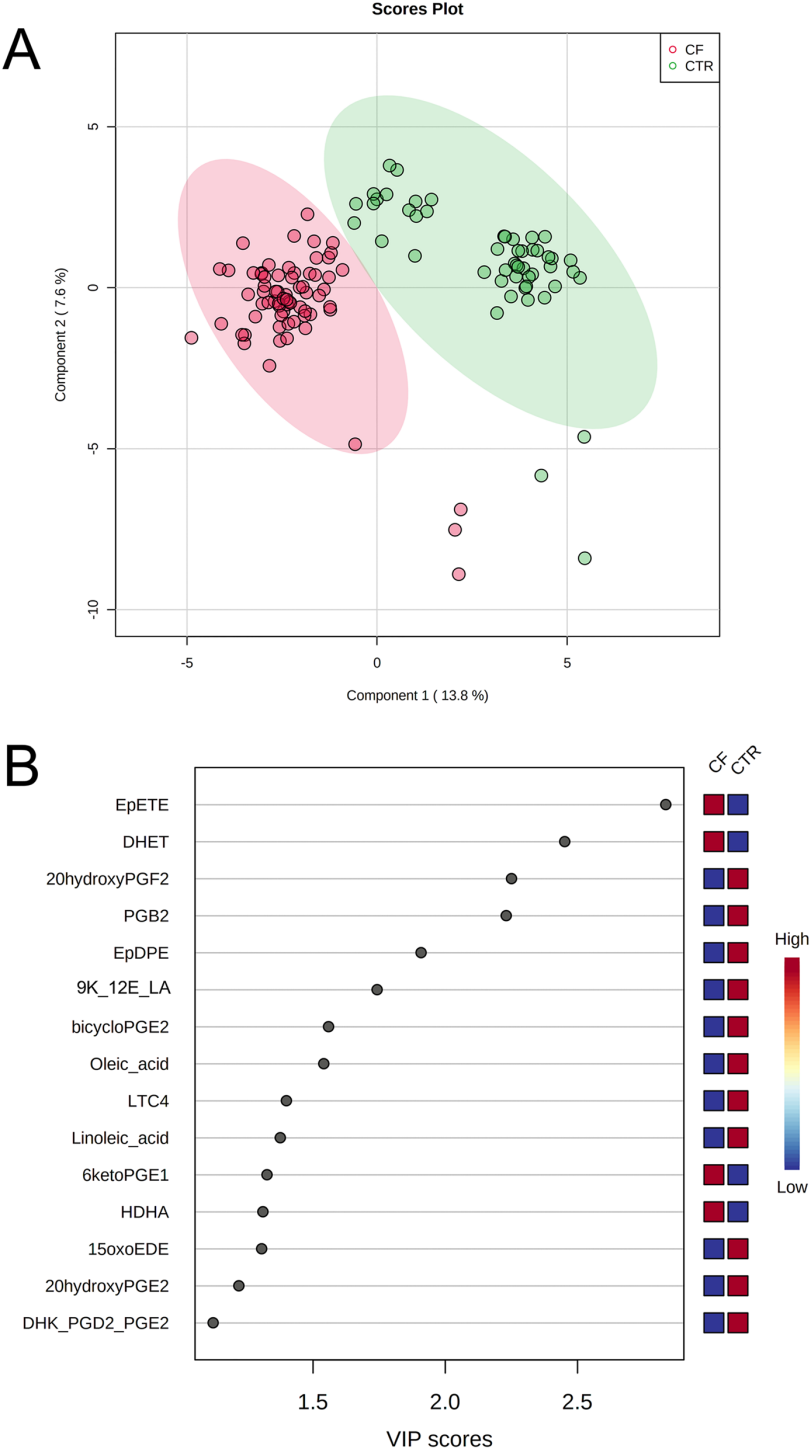
PLS-DA analysis discriminating patients with CF and controls (CTR). (**A**) 2D score plot; (**B**) VIP score of the first 15 features.CF: cystic fibrosis; PLS-DA: partial least-squares discriminant analysis.

PLS-DA 2D score plot showed that the first and the second components (PC1 and PC2) separate completely the CF patients from CTR, although 4 CF and 3 CTR are positioned outside their respective clusters (Fig. 1A). PC1 and PC2 explain the 21.4% of model variance. Figure 1B showed the VIP score of the first 15 variables in the PLS-DA model. Among these, EpETE, DHET, 6ketoPGE1 and HDHA were significantly higher in saliva from patients with CF than in saliva from CTR. While 20-hydroxyPGF2A, PGB2, EpDPE, 9K-12E-LA, bicycloPGE2, oleic acid, LTC4, linoleic acid, 15oxoEDE, 20 hydroxyPGE2 and DHK-PGD2-PGE2 resulted significantly lower in saliva from patients with CF than in CTR.

**Figure 2:**
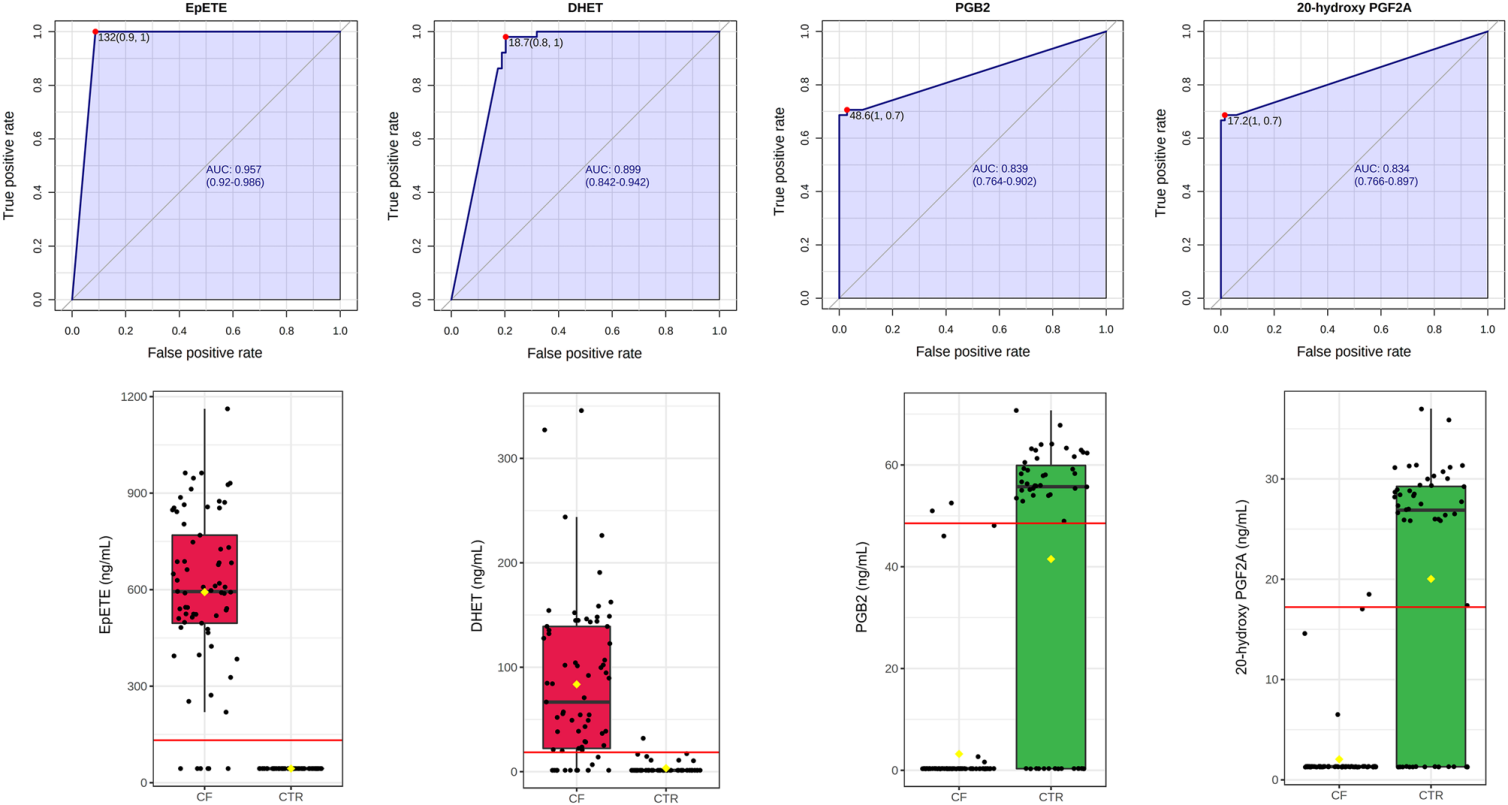
Univariate ROC curve analysis for EpETE, DHET, PGB2, and 20-hydroxyPGF2A. Red lines in box plots represent the best cut-off values.

Then, we performed a classical univariate ROC curve analysis for the first 4 variables with a VIP score higher than 2, i.e., EpETE, DHET, 20-hydroxyPGF2A, and PGB2. The area under the ROC curve (AUC) ranged from 0.834 for 20-hydroxyPGF2A to 0.957 for EpETE (Fig. 2). The box plots shows that some CF patients had the levels of these metabolites similar to those in controls. In particular, 3/69 patients had DHTE, PGB2 and 20-hydroxy-PGF2A levels that shared those in controls, and other 2 patients for EpETE and DHET. These 5 patients had an age ranging from 20 to 43 years and 3/5 are males. In addition, 4/5 patients had a mild lung disease (FEV1 > 69%) without *P. aeruginosa* (PA) colonization and pancreatic insufficiency, although 2 patients had inferior turbinate hypertrophy (ITH). The demographic and clinical parameters of all patients and controls are reported in Table 1.

**Table 1:** Demographic and clinical parameters of patients with cystic fibrosis (CF) and healthy subjects (controls).

	**CF patients** (n = 69)	**Controls** (n = 50)	P value
Age (years)	30 (22–40)	33 (29–41)	n.s
Males, n (%)	37 (53.6)	18 (36)	n.s
Lung disease severity, n (%):			
Severe ^a^	7 (10.2)	-	
Moderate ^b^	21 (30.4)	-	
Mild ^c^	41 (59.4)	-	
Pancreatic insufficiency, n (%)	37 (53.6)	-	
PA colonization, n (%)	34 (49.3)	-	
ITH, n (%)	10 (14.5)	-	
NP, n (%)	9 (13.0)	-	

^a^ FEV1 < 40%; ^b^ FEV1 40–69%; ^c^ FEV1 > 69%. ITH: inferior turbinate hypertrophy; NP: nasal polyposis; n.s.: not significant; PA: *P. aeruginosa.*

**Figure 3:**
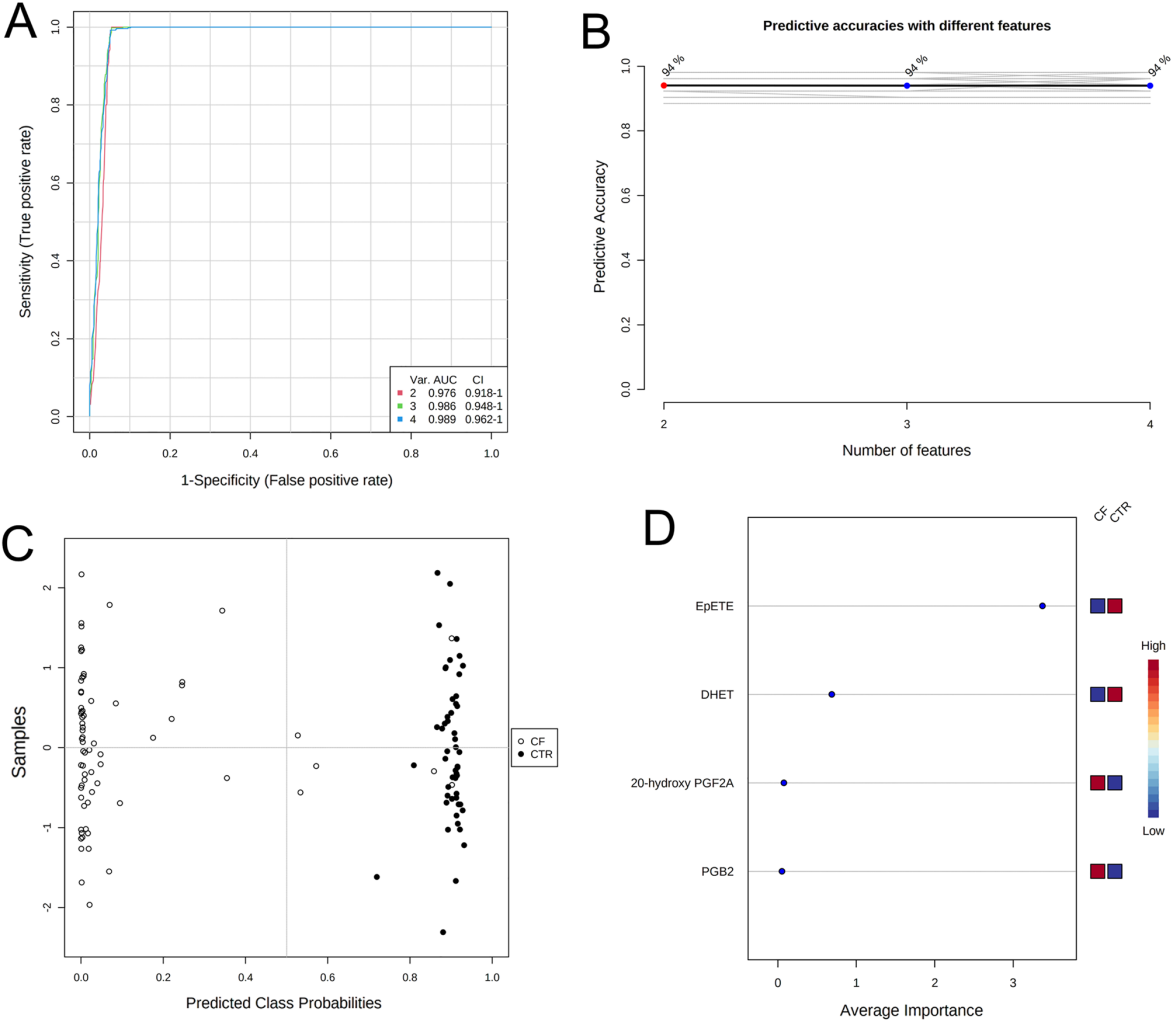
Multivariate ROC curve based exploratory analysis. (**A**) comparison of ROC curves to discriminate patients with CF from controls (CTR) using from 2 to 4 variables; (**B**) predictive accuracies of models from 2 to 4 variables; (**C**) predicted class probabilities for each sample using the 4 features-model; (**D**) rank features for average importance. AUC: area under the ROC curve; CF: cystic fibrosis; ROC: receiver operating characteristic.

Then, we explored a multivariate ROC curve based exploratory analysis using all the 4 variables. Figure 3A shows the ROC curves based on the cross-validation performance. The AUC ranged from 0.976 for the 2 features-model to 0.989 for the 4 features-model. According to the criteria of Jones and Athanasiou^22^, the 4 features-model shows an “excellent” AUC. The predictive accuracies were 94% for all three models (Fig. 3B). Figure 3C shows the predicted class probabilities (average of the cross-validation) for each sample using the best classifier, i.e., the 4 features-model. The confusion matrix showed that the 4 features-model has a sensitivity of 91.3% and a specificity of 100%. The rank features shows that EpETE presents the higher average importance in the 4 features-model (Fig. 3D).

**Figure 4:**
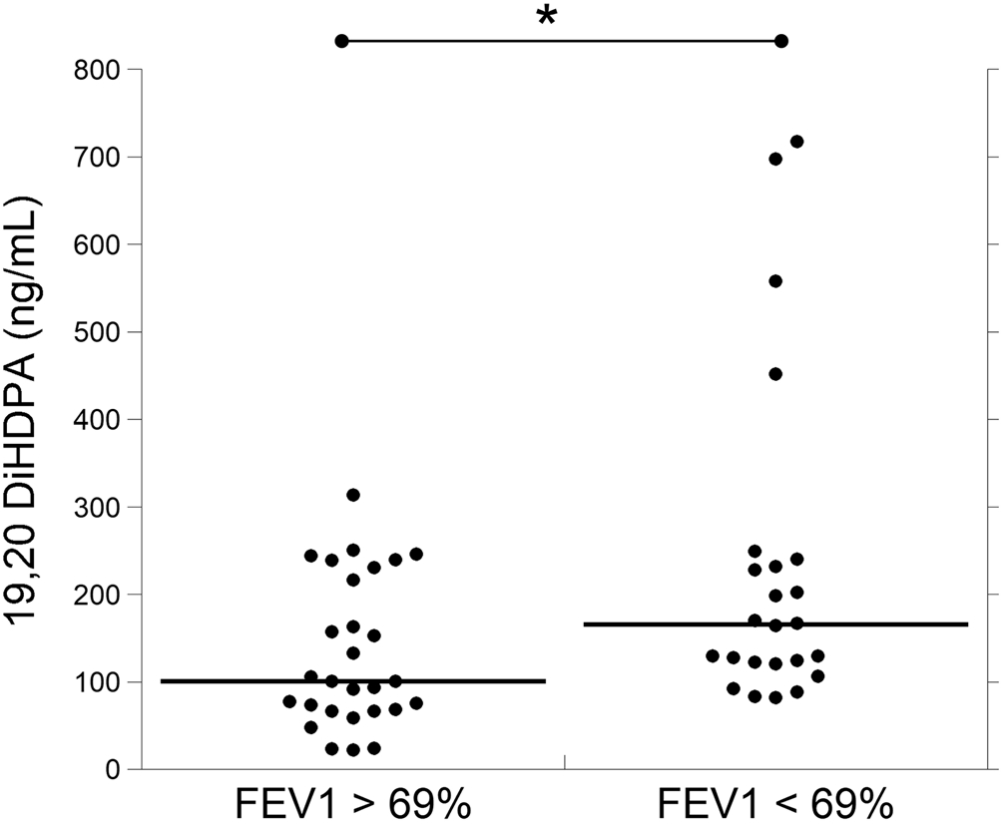
Comparison of salivary levels of 19,20 DiHDPA between CF patients with FEV1 > 69% (n = 41) and FEV1 < 69% (n = 28). DiHDPA was not detectable (< 0.1 ng/mL) in 13 patients with FEV1 > 69% and in 4 patients with FEV1 < 69%. FEV1: forced expiratory volume in the 1st second. *p < 0.01.

Finally, we related all oxylipin levels to clinical parameters of patients with CF, i.e., severity of the pulmonary disease (evaluated by FEV1%), pancreatic status (sufficiency versus insufficiency), presence or absence of chronic colonization by PA and presence or absence of ITH and nasal polyposis (NP) (Table 1). None of the biochemical markers correlated with none of the clinical parameters, except for salivary levels of 19,20 DiHDPA. This oxylipin resulted significantly higher (p < 0.01) in patients with a FEV1 < 69% (moderate/severe pulmonary disease), in comparison to patients with a FEV1 > 69% (mild pulmonary disease, Fig. 4).

**Figure 5:**
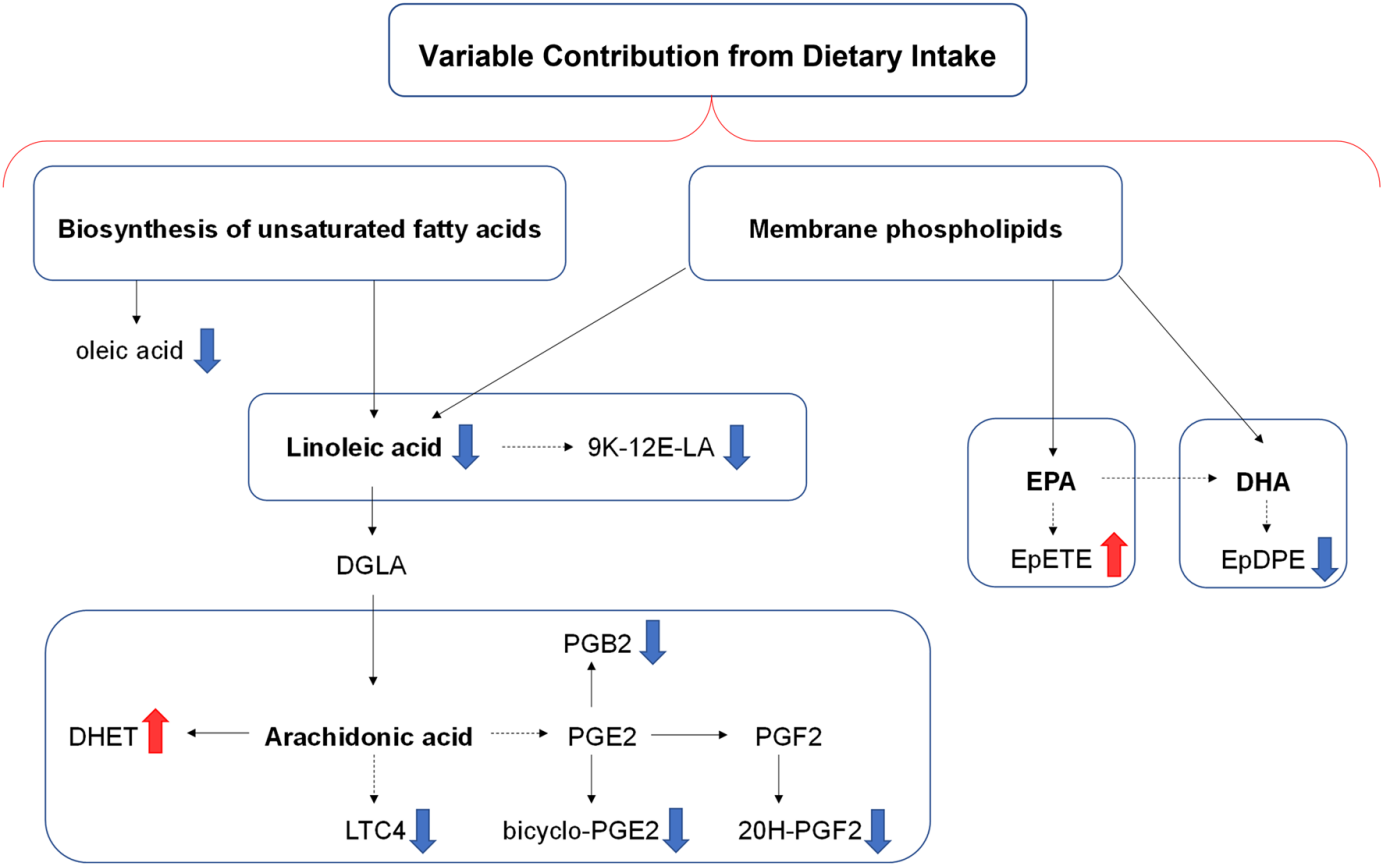
Simplified KEGG maps of the top 10 differentially expressed oxylipins and fatty acids in CF patients. Blue and red arrows mean down-regulated and up-regulated, respectively. 9 K-12E-LA: 9 keto-12 epoxy-octadecenoic acid; DGLA: dihomo-γ-linolenic acid; DHA: docosahexaenoic acid; DHET: dihydroxy-eicosatrienoic acids; EPA: eicosapentaenoic acid; EpDPE: epoxy-docosapentanoic acid; EpETE: epoxy-eicosatetraenoic acids; LTC4: leukotriene C4; PGB2: prostaglandin B2; PGE2: prostaglandin E2; PGF2: prostaglandin F2α.

Figure 5 shows the metabolic pathways involved in biosynthesis of the top 10 differentially expressed oxylipins/fatty acids in CF patients with a VIP score higher or equal to 1.5 (Fig. 1B). Five out of the top 10 molecules belong to the arachidonic acid pathway.

## Discussion

We observed a strongly different profile of oxylipins and fatty acids in saliva from patients with CF as compared to healthy subjects matched for age and gender. The levels of most molecules resulted significantly different, and among these, 15 molecules had a high VIP score in discriminating the two conditions. In particular, in saliva from patients with CF, we observed an increase of average values of EpETE, i.e., epoxy-eicosatetraenoic acids. They derive from eicosapentaenoic acid (EPA) in a reaction catalyzed by CYP450 epoxygenases. 17,18-EpETE displays a bronchodilator effect helping the release of tracheal smooth muscle in guinea pig^23^. Furthermore, it causes a relaxing effect on the smooth muscle of distal airways and arterial vessels at pulmonary level^24^. EpETE also displays an anti-inflammatory activity inhibiting TNFα-induced inflammation in human airway cells and reducing murine airway inflammation induced by LPS^25^, although we found a positive correlation between EpETE and IL-6. The average levels of DHET, i.e., dihydroxy-eicosatrienoic acids, were significantly increased in turn in saliva from patients with CF. They are synthesized from EET (epoxy-eicosatrienoic acid) in a reaction catalyzed by soluble epoxide-hydrolase. Recently, the role of 14,15 DHET in protecting vascular endothelia upregulating endothelial NO synthase expression^26^, and the immunomodulatory activity of this molecule has been described^27^. In addition, in saliva from patients with CF we found an increase of 6-keto-PGE1 together with a negative correlation *versus* TNFα. This molecule is mainly produced from the hydrolysis of PGI2 by the platelets for the effect of the enzyme 9 hydroxy-prostaglandin dehydrogenase. 6-keto-PGE1 is more stable to the degradation at pulmonary level^28^ and presents a higher bronchodilator effect as compared to PGI2^29^. Furthermore, it inhibits platelet aggregation in small intrapulmonary vessels and reduces vascular pulmonary resistance^30^. Finally, among molecules significantly increased in saliva from patients with CF, we found HDHA (hydroxy-docoexanoic acid), a member of specialized pro-resolving mediator family. It is synthesized by endothelial cells and acts as a modulator of vascular damage^31^. Furthermore, it attenuates the effects of leukocytes stimulated by cytokines^32^ that are significantly increased in saliva from CF patients^20^. Interestingly, HDHA was recently used in the therapy of chronic pain^33^.

On the other hand, we observed a significant reduction of other oxylipins in saliva from patients with CF as compared to healthy controls. Among these oxylipins, we found PGB2 that is a powerful vascular^34^ and pulmonary constrictor^35^ and is involved in the pathogenesis of pulmonary hypertension^36^. EpDPE, i.e., epoxy-docosapentanoic acid, that is synthesized from DHA for the effect of CYP450 and has a bronchoconstrictor effect in turn^37^. LTC4, that causes bronchoconstriction with a more powerful effect than histamine, as demonstrated also by inhalation in healthy volunteers^38^. LTC4, that is synthesized from LTA4 and in turn is a powerful bronchoconstrictor^39^, and is involved in modulating mucus secretion, inflammation, and tissue remodeling^40^. In addition, we found a significant reduction of both oleic and linolenic acid in saliva from patients with CF as compared to healthy controls. Both these fatty acids are strong modulators of inflammation, even if a large body of literature described contrasting effect of these molecules^41,42^.

Overall, the pattern of oxylipins and fatty acids that we found in saliva from patients with CF seems to be addressed toward an anti-inflammatory response with an increase of molecules that reduce bronchoconstriction and vascular resistance which are the hallmark of CF chronic inflammation^8,43,44^ and a reduction of pro-inflammatory molecules, mainly belonging to the arachidonic acid metabolism. It is difficult define if these changes would depend on the enhanced synthesis of pro-resolving molecules and the down regulated synthesis of pro-inflammatory ones or on the consumption of proinflammatory molecules. Interestingly, the levels of salivary oxylipins were not related to bacterial colonization or to sinunasal inflammation, although we found DHTE, PGB2 and 20-hydroxy-PGF2A levels similar to the controls in four patients with a mild lung disease and without NP and PA colonization. Overall, these findings suggest that the novel equilibrium between these molecules found in saliva from CF patients would be a long-standing “readaptation” aimed to contrast chronic inflammation.

Previous studies performed on BAL, sputum or pulmonary exhalated of patients with CF showed an unbalance between pro-resolving/pro-inflammatory markers in most cases in favor of the lasts^12,45^. Other studies described a significant reduction of proinflammatory molecules like Lipoxin A4 in sputum from patients with CF^46,47^, while in the present study the level of such molecule in saliva from patients with CF was not significantly different in comparison to healthy subject. Finally, enhanced levels of molecules with pro-resolving activity, among which epoxy-fatty acids, were reported^48^ in agreement with our results.

The main limit of this and previous studies is that we reported the data of a single observation of oxylipin levels in patients with CF. While a dynamic and perspective analysis of such biomarkers during a long-standing follow-up of patients with CF would help to better understand the role of these molecules in the pathogenesis of the disease and the possible clinical use of targeted therapies based on the modulation of specific oxylipin synthesis and degradation^48^ or on the direct use of pro-resolving molecules^16,17,49^. In this perspective, resting saliva is more suitable than sputum, which is more difficult to collect in small children although less invasive than BAL. In fact, our group already analyzed cytokines in saliva from patients with CF demonstrating a correlation between IL-6 and IL-8 with the sinunasal inflammation and an inverse correlation between TNF-alpha and the severity of lung diseases^20^, and more recently we demonstrated a correlation between unsaturated/saturated fatty acid ratio in saliva and the severity of the lung disease in patients with CF^5^.

To conclude: the study of oxylipins provides a great impulse to the knowledge of the pathogenesis of lung inflammation in patients with CF offering the opportunity to define prognostic biomarkers and to reveal and monitor novel targeted therapeutic approaches. Resting saliva is an easy, rapid, and poorly invasive approach to effectively analyze oxylipins.

## Methods

### Study population

The study, performed accordingly to current version of the Helsinki Declaration, was approved by the Ethical Committee of the University of Naples Federico II (Protocol # 77-21). Informed consent was obtained from all subjects involved in the study. Sixty-nine patients with CF were randomly recruited at the CF Regional Centre of Campania Region, while as a control group we recruited 50 healthy volunteers, age and sex matched with CF patients. The diagnosis of CF was performed according to the guidelines of the CF Foundation^50^. Gene sequencing was performed to define the *CFTR* genotype^51,52^. Sweat test was performed using standard guidelines after pilocarpine iontophoresis^53^. Pancreatic sufficiency was defined on the basis of at least two values of faecal pancreatic elastase higher than 200 μg/g measured outside acute gastrointestinal diseases^54^. Sinusitis, NTH and NP were diagnosed in accordance with chest computed tomography (CT) findings. At least two measures of forced expiratory volume in the 1st second (FEV1), expressed as percentage of predicted value for age, according to standardized reference equations for spirometry^55^ were recorded in the day of saliva collection. According to forced expiratory volume (FEV)1 percentage values, pulmonary disease was classified as mild (FEV1 > 69%), moderate (FEV1 40–69%) and severe (FEV1 < 40%)^56^. Endobronchial bacterial infections were detected by sputum or hypopharyngeal aspirate. *Pseudomonas aeruginosa* (PA) chronic infection was defined following modified Leeds criteria^57^.

### Samples

We collected resting saliva samples (i.e., without stimulation) in the morning (9–12 a.m.), after two hours at least of fasting, from all CF patients and controls. Resting saliva (about 1–3 mL) was collected in sterile plastic tubes in ice and then centrifuged for 30 min at 14,000 g to remove bacteria/cellular debris. In order to prevent in vitro auto-oxidation processes^58^, the supernatants were spiked of butylated hydroxytoluene, at the final concentration of 1 mg/mL, and stored at -80 °C until analysis.

### Extraction of target oxylipins from saliva samples

Two-hundred microliters of cold methanol were added to 50 µl of saliva samples and incubated for 30 min in order to precipitate the protein fraction interfering with the analysis of the metabolites. The samples were centrifuged at 12,000 rpm for 10 min and the supernatants were dried and subsequently resuspended in 50 µl of methanol before the LC-MRM/MS analysis.

Two mixtures of linoleic acid oxylipins (Cay20794-1, Cayman chemicals) and EPA Oxylipin (Cay21393, Cayman chemicals) were mixed at 5000 ng/ml and diluted in range of 1000 to 0.1 ng/ml in methanol and used for the quantitative analysis of target molecules included in the method.

### LC-MRM/MS analysis

Saliva extracts and standard mixtures were analyzed by using liquid chromatography combined to 5500 QTRAP (AB Sciex) mass spectrometer. The separation was carried out on a Kinetex column 5 µm C18 100 Å (100 × 2.1 mm) at temperature of 40 °C at a flow rate of 0.2 mL/min. An aqueous solution acidified with 0.5% acetic acid was used as solvent A while 50% Acetonitrile, 50% Isopropanol, 0.5% Acetic Acid solution was used as a solvent B. A gradient was set according to the following parameters: 0–3 min 5% B, 3–9 min 95% B, 9–10 min 95% B, 10–10.5 min 5% B along a chromatographic run of 10.5 min duration. The parameters of the electrospray source were: voltage of -4500 V, source temperature of 500° C, curtain gas at 20 psi, ion source gas 1 at 60 psi, and ion source gas 2 at 60 psi. The analyses were performed in negative ion polarity by using MRM transitions and parameters summarized in Supplementary Table 4.

### Inflammation biomarkers

Serum CRP was measured by a commercial kit (Abbott Diagnostics, Rome, Italy) with an automated biochemistry analyzer (Architect ci 16,200 Integrated System, Abbott Diagnostics, Rome, Italy). Salivary cytokines were analyzed using human IL-6, IL-8, and TNFα ELISA Max™ Set Deluxe kits (BioLegend, Inc., San Diego, CA, USA), as previously described^20^.

### Statistical analysis

Continuous data are reported as median and interquartile range (25°-75° percentiles) and compared by Mann–Whitney U test. The Shapiro–Wilk test was applied to evaluate the normality of distributions. Categorical data were reported as frequency (percentage) and compared by chi-square test. Correlations between variables were evaluated using Spearman correlation analysis. Principal Component Analysis (PCA) was applied to detect sample metabolite trends and clustering in an unsupervised manner, and the Partial Least-Squares Discriminant Analysis (PLS-DA) was then performed to reinforce classification and to better identify clustering. Before PCA and PLS-DA, data were batch normalized dividing each variable of each group by the median of all original values of that group. Finally, the dataset was log transformed and auto-scaled. Receiver operating characteristic (ROC) curve analysis was used and areas under the curve (AUC) were calculated to compare the effectiveness of different molecules to discriminate CF patients from controls. We used linear SVM classification and SVM built-in as feature ranking method. According to the criteria of Jones and Athanasiou^22^, AUC > 0.97, 0.93–0.96, 0.75–0.92, and 0.6–0.74 were interpreted as “excellent,” “very good,” “good,” and “reasonable,” respectively. Statistical analysis was performed by SPSS (version 27, IBM SPSS Statistics) and MetaboAnalyst 5.0 online package[https://www.metaboanalyst.ca].

Variables were mapped to Kyoto Encyclopedia of Genes and Genomes (KEGG) pathway database^59,60^[https://www.genome.jp/kegg/pathway.html#metabolism]. Graphics have been performed by KaleidaGraph software (version 4.5.4, Synergy, Reading, PA, USA). P values < 0.05 were considered as significant.

## Supplementary Information

Supplementary Information.

## Data Availability

The datasets used and/or analysed during the current study are available from the corresponding author on reasonable request.

## Abbreviations

CF: Cystic fibrosis
BAL: Bronchoalveolar lavage
FEV: Forced expiratory volume
NP: Nasal polyposis
Pa: *Pseudomonas aeruginosa*
PCA: Principal component analysis
PLS-DA: Partial least-squares discriminant analysis
ROC: Receiver operating characteristic
AUC: Area under curve
