# Supplementary material for: Oxylipin profile in saliva from patients with cystic fibrosis reveals a balance between pro-resolving and pro-inflammatory molecules
Source: Sci Rep. 2022 Apr 7;12:5838. doi: 10.1038/s41598-022-09618-7 (PMC8991203; doi:10.1038/s41598-022-09618-7)
Supplement: Supplementary file 1 — Supplementary Information. [file 41598_2022_9618_MOESM1_ESM.docx]

**Supplementary Table 1.** Comparison of salivary oxylipins and total fatty acids between patients with cystic fibrosis (CF) and healthy subjects (controls).

|  | **CF (n = 69)** | | |  | **CONTROLS (n = 50)** | | | |  |
| --- | --- | --- | --- | --- | --- | --- | --- | --- | --- |
| **Oxylipins (ng/mL)** | Median | Percentiles |  |  | Median | | Percentiles | | **P value*** |
|  |  | 25° | 75° |  |  | | 25° | 75° |  |
| Adrenic acid | 498.14 | 356.59 | 786.24 |  | 1077.42 | | 1041.18 | 1127.59 | **< 0.0001** |
| 20-COOH-AA | 138.48 | 82.63 | 235.64 |  | 158.49 | | 150.05 | 166.17 | 0.7905 |
| EPA | 12.20 | 8.89 | 15.35 |  | 5.27 | | 4.97 | 6.12 | **0.0010** |
| bicyclo-PGE2 | 5.34 | 3.33 | 5.61 |  | 3.05 | | 2.88 | 3.26 | **0.0012** |
| Nitroleate | 105.70 | 87.84 | 154.90 |  | 105.05 | | 95.29 | 113.48 | 0.3778 |
| HDHA | 84.66 | 59.94 | 145.02 |  | 51.64 | | 47.89 | 55.95 | **< 0.0001** |
| HDoHE | 97.18 | 77.10 | 152.58 |  | 86.11 | | 80.56 | 91.77 | **0.0178** |
| HEPE | 437.42 | 328.42 | 651.03 |  | 279.53 | | 258.57 | 300.21 | **< 0.0001** |
| HETE_1 | 137.49 | 113.94 | 246.32 |  | 115.55 | | 104.00 | 122.74 | **< 0.0001** |
| HETE_2 | 162.65 | 128.91 | 245.95 |  | 113.80 | | 108.89 | 124.90 | **< 0.0001** |
| HETrE | 21.15 | 16.21 | 33.17 |  | 19.53 | | 18.65 | 21.27 | 0.4840 |
| 9K-12-E-LA | 106.42 | 82.43 | 171.50 |  | 799.41 | | 103.03 | 866.43 | **< 0.0001** |
| KDPA | 1265.74 | 1143.17 | 1566.93 |  | 1033.94 | | 985.91 | 1121.11 | **< 0.0001** |
| KODE | 20.26 | 11.06 | 34.84 |  | 27.75 | | 26.04 | 30.34 | **0.0405** |
| HODE | 162.30 | 124.23 | 210.82 |  | 122.92 | | 116.57 | 129.93 | **< 0.0001** |
| HOTrE | 3741.83 | 2509.81 | 4997.86 |  | 5305.83 | | 4876.20 | 5593.84 | **< 0.0001** |
| HXA3/HXB3 | 19.82 | 15.11 | 25.97 |  | 34.30 | | 31.41 | 36.24 | **0.0096** |
| DHET | 94.68 | 40.96 | 144.39 |  | 14.62 | | 10.96 | 17.34 | **< 0.0001** |
| diHETE | 156.42 | 113.42 | 221.15 |  | 129.44 | | 121.39 | 143.49 | **0.0095** |
| DiHETrE | 69.59 | 42.44 | 100.27 |  | 68.89 | | 63.64 | 74.07 | 0.6256 |
| DiHOME | 312.43 | 284.41 | 461.56 |  | 203.27 | | 185.12 | 218.83 | **< 0.0001** |
| oxoETE | 70.78 | 49.14 | 113.83 |  | 49.05 | | 45.72 | 52.42 | **< 0.0001** |
| TriHOME | 334.47 | 273.12 | 440.17 |  | 218.84 | | 209.71 | 235.24 | **< 0.0001** |
| oxoODE | 195.11 | 159.09 | 278.37 |  | 184.50 | | 171.05 | 202.85 | 0.1108 |
| EDP | 123.40 | 85.95 | 206.24 |  | 83.63 | | 78.63 | 88.90 | **< 0.0001** |
| EEQ | 58.36 | 39.85 | 83.67 |  | 49.16 | | 45.44 | 51.52 | **0.0194** |
| EET | 81.89 | 66.57 | 126.59 |  | 94.47 | | 86.93 | 100.83 | 0.2809 |
| EpDPE | 57.24 | 41.33 | 99.38 |  | 1126.57 | | 358.88 | 1213.56 | **< 0.0001** |
| EpETrE | 26.07 | 13.21 | 32.78 |  | 25.50 | | 23.40 | 26.85 | 0.7517 |
| EpOME | 268.31 | 227.03 | 397.60 |  | 278.54 | | 262.25 | 298.50 | 0.8471 |
| EpETE | 608.12 | 519.14 | 842.22 |  | < 0.5 | |  |  | **< 0.0001** |
| 10(S),17(S)-DiHDoHE | 52.19 | 27.36 | 94.81 |  | 19.00 | | 17.93 | 20.03 | **< 0.0001** |
| 19,20-DiHDPA | 129.51 | 82.31 | 231.35 |  | 59.32 | | 57.65 | 66.00 | **< 0.0001** |
| 15-oxoEDE | 48.08 | 38.98 | 72.35 |  | 54.33 | | 53.16 | 56.91 | **0.0485** |
| 12S-HHTrE | 45.82 | 37.00 | 64.69 |  | 62.76 | | 58.31 | 67.99 | **0.0184** |
| 11H-9E-LA | 640.07 | 573.85 | 790.41 |  | 498.58 | | 463.62 | 545.98 | **< 0.0001** |
|  |  |  | | |  |  |  |  |  |
|  | **CF** | | |  | **CONTROLS** | | | |  |
| **Oxylipins (ng/mL)** | Median | Percentiles |  |  | Median | | Percentiles |  | **P value*** |
|  |  | 25° | 75° |  |  | | 25° | 75° |  |
| 9-HpODE | 55.20 | 51.49 | 59.41 |  | 51.80 | | 48.73 | 54.78 | **0.0009** |
| 7(R) Maresin-1 | 15.97 | 12.07 | 28.09 |  | 6.20 | | 5.98 | 6.50 | **< 0.0001** |
| Protectin D1 | 37.55 | 19.16 | 80.02 |  | 38.80 | | 36.00 | 42.24 | 0.6963 |
| Resolvin D | 150.74 | 112.81 | 268.22 |  | 159.67 | | 151.38 | 174.38 | 0.6608 |
| dihomo PGE2 | 932.71 | 748.78 | 1250.35 |  | 661.40 | | 628.95 | 685.06 | **< 0.0001** |
| PDX (10S,17S-DiHDHA) | 27.47 | 15.90 | 42.01 |  | 13.66 | | 12.87 | 14.35 | **< 0.0001** |
| PGB2 | 47.08 | 2.43 | 51.37 |  | 58.22 | | 55.39 | 62.41 | **< 0.0001** |
| PGE1/PGD1 | 39.26 | 34.46 | 55.87 |  | 68.39 | | 54.78 | 75.81 | **< 0.0001** |
| PGE2/PGD2 | 1240.30 | 792.05 | 1843.90 |  | 685.05 | | 650.26 | 724.12 | **< 0.0001** |
| PGE3/PGD3 | 233.85 | 200.28 | 286.80 |  | 517.24 | | 492.50 | 564.82 | **< 0.0001** |
| PGJ2 | 28.37 | 22.37 | 37.46 |  | 22.93 | | 21.19 | 25.98 | **0.0021** |
| PGF2 | 12.81 | 9.68 | 19.65 |  | 12.39 | | 11.21 | 12.89 | 0.3600 |
| 6-keto PGE1 | 7.91 | 4.61 | 10.86 |  | 5.56 | | 5.23 | 6.34 | 0.2500 |
| 15-keto PGE2 | 34.22 | 25.64 | 49.50 |  | 38.21 | | 35.25 | 40.50 | 0.0843 |
| 1,1-dihomo-PGF2 | 45.93 | 34.70 | 60.91 |  | 44.62 | | 41.43 | 46.53 | 0.5515 |
| 20-hydroxy PGE2 | 23.06 | 17.28 | 30.61 |  | 66.12 | | 34.83 | 72.90 | **< 0.0001** |
| 20-hydroxy PGF2 | 15.83 | 8.55 | 18.17 |  | 28.54 | | 26.63 | 30.34 | **< 0.0001** |
| DHK PGD2/PGE2 | 5.33 | 3.96 | 7.79 |  | 3.75 | | 3.58 | 4.04 | **0.0003** |
| DHK PGF/PGD | 14.02 | 6.52 | 20.97 |  | 19.62 | | 18.43 | 20.64 | **0.0023** |
| LTB4 | 202.91 | 175.62 | 237.68 |  | 188.54 | | 179.37 | 197.83 | 0.0616 |
| LXA5 | 25.27 | 15.75 | 35.01 |  | 32.30 | | 30.31 | 34.25 | **0.0034** |
| LXB4 | 69.79 | 54.69 | 110.61 |  | 39.44 | | 35.61 | 40.75 | **< 0.0001** |
| LTC4 | 17.70 | 7.87 | 29.50 |  | 22.21 | | 21.12 | 23.52 | 0.4860 |
| LTD4 | 8.23 | 4.93 | 14.46 |  | 10.97 | | 10.54 | 11.94 | 0.4135 |
| LTE4 | 17.19 | 5.07 | 277.70 |  | 859.98 | | 818.40 | 899.04 | **< 0.0001** |
| LXA4 | 7552.69 | 4347.47 | 11337.82 |  | 6053.03 | | 5538.50 | 6463.79 | **0.0358** |
| 12oxo LTB4 | 14.49 | 8.84 | 26.27 |  | 96.72 | | 89.62 | 102.98 | **< 0.0001** |
| 20-carboxy LTB4 | 93.10 | 75.35 | 133.86 |  | 138.25 | | 128.90 | 149.85 | **< 0.0001** |
| 20-hydroxy LTB4 | 2750.06 | 2420.50 | 3402.61 |  | 2442.43 | | 2333.61 | 2631.94 | **0.0005** |
| **Total fatty acids (µg/mL)** |  |  |  |  |  | |  |  |  |
| Palmitic acid | 363.60 | 93.44 | 580.28 |  | 501.44 | | 317.74 | 664.18 | 0.0867 |
| Stearic acid | 486.97 | 215.90 | 993.71 |  | 634.07 | | 321.98 | 772.24 | 1.0000 |
| Oleic acid | 20.59 | 7.17 | 58.49 |  | 79.16 | | 61.14 | 125.11 | **< 0.0001** |
| Linoleic acid | 6.67 | 3.77 | 12.65 |  | 25.01 | | 7.25 | 59.03 | **< 0.0001** |

* Mann-Whitney U test, significant P values are reported in bold.

**Supplementary Table 2.** Comparison of serum hs-CRP and salivary cytokines between patients with cystic fibrosis (CF) and healthy subjects (controls).

|  | **CF (n = 69)** | | |  | **Controls (n = 50)** | | |  |
| --- | --- | --- | --- | --- | --- | --- | --- | --- |
|  | Median | Percentiles |  |  | Median | Percentiles |  | **P value*** |
|  |  | 25° | 75° |  |  | 25° | 75° |  |
| hs-CRP (mg/dL) | 0.34 | 0.33 | 0.69 |  | < 0.33 |  |  | **< 0.0001** |
| IL-6 (pg/mL) | 58.2 | 40.4 | 88.6 |  | 21.6 | 7.1 | 40.8 | **< 0.0001** |
| IL-8 (pg/mL) | 297 | 123 | 488 |  | 52.8 | 29.6 | 80.7 | **< 0.0001** |
| TNFα (pg/mL) | 28.2 | 15.7 | 53.1 |  | 11.1 | 6.6 | 29.1 | **0.002** |

* Mann-Whitney U test, significant P values are reported in bold. hs-CRP: high sensitivity C-reactive protein; IL: interleukin; TNFα: tumor necrosis factor α.

**Supplementary Table 3.** Spearman correlations analysis of serum hs-CRP and salivary cytokines *versus* oxylipins in patients with cystic fibrosis (CF).

|  | **P value** | **r_s_** |
| --- | --- | --- |
| Serum hs-CRP *vs* |  |  |
| EET | 0.039 | -0.267 |
| **EpDPE** | 0.049 | -0.274 |
| 6keto-PGE1 | 0.038 | -0.342 |
| LTD4 | 0.010 | -0.438 |
| Salivary IL-6 *vs* |  |  |
| **KODE** | 0.006 | -0.556 |
| **EpETE** | 0.001 | 0.487 |
| **LTE4** | 0.002 | -0.905 |
| Salivary IL-8 *vs* |  |  |
| **HOTrE** | 0.001 | 0.506 |
| **HXA3/HXB3** | 0.039 | -0.355 |
| oxoODE | 0.037 | 0.323 |
| **EDP** | 0.003 | -0.464 |
| **11H-9E-LA** | 0.004 | -0.434 |
| Resolvin D | 0.005 | -0.432 |
| **PGE2/PGD2** | 0.047 | 0.329 |
| **LXA5** | 0.004 | 0.474 |
| **LXA4** | 0.035 | 0.343 |
| Salivary TNFα *vs* |  |  |
| **HETE** | 0.032 | -0.335 |
| **9K-12E-LA** | 0.043 | -0.318 |
| **HXA3/HXB3** | 0.001 | -0.554 |
| Resolvin D | 0.023 | -0.358 |
| **PGE1/PGD1** | 0.038 | -0.330 |
| **6keto-PGE1** | 0.003 | -0.600 |
| **DHX PGD2/PGE2** | 0.041 | -0.552 |
| LTB4 | 0.001 | -0.555 |
| LTD4 | 0.001 | -0.656 |
| **20-carboxy-LTB4** | 0.026 | -0.398 |

Differentially expressed oxylipins in CF patients are reported in bold. hs-CRP: high sensitivity C-reactive protein; IL: interleukin; TNFα: tumor necrosis factor α.

**Supplementary Table 4.** MRM transitions and parameters of LC-MRM/MS analysis

| **ID** | **Q1** | **Q3** | **Dwell time** | **DP** | **CE** | **CXP** |
| --- | --- | --- | --- | --- | --- | --- |
| 12S-HHTrE | 279.2 | 261 | 50 | -21 | -18 | -17 |
| 12S-HHTrE | 279.2 | 217 | 50 | -21 | -18 | -17 |
| 12S-HHTrE | 279.2 | 179.2 | 50 | -21 | -18 | -17 |
| 13-HOTrE | 293.2 | 195 | 50 | -80 | -24 | -15 |
| 13-HOTrE | 293.2 | 205 | 50 | -80 | -24 | -15 |
| 13-HOTrE | 293.2 | 277 | 50 | -80 | -24 | -15 |
| 13-KODE | 293.2 | 113.1 | 50 | -70 | -29 | -15 |
| 13-KODE | 293.2 | 167 | 50 | -70 | -20 | -15 |
| 13-oxoODE | 293.2 | 195.1 | 50 | -120 | -28 | -13 |
| 13-oxoODE | 293.2 | 113 | 50 | -120 | -28 | -13 |
| 13-oxoODE | 293.2 | 177 | 50 | -120 | -28 | -13 |
| 9-HOTrE | 293.2 | 171.1 | 50 | -105 | -22 | -11 |
| 9-HOTrE | 293.2 | 185 | 50 | -105 | -22 | -11 |
| 9-HOTrE | 293.2 | 277 | 50 | -105 | -22 | -11 |
| 9-KODE | 293.2 | 185.2 | 50 | -70 | -18 | -16 |
| 9-oxoODE | 293.2 | 185.1 | 50 | -110 | -28 | -15 |
| 9-oxoODE | 293.2 | 125 | 50 | -110 | -28 | -15 |
| 9-oxoODE | 293.2 | 149 | 50 | -110 | -28 | -15 |
| 12(13)-EpOME | 295.2 | 195.2 | 50 | -50 | -23 | -9 |
| 12,13-EPOME | 295.2 | 195 | 50 | -65 | -26 | -9 |
| 12,13-EPOME | 295.2 | 113 | 50 | -65 | -26 | -9 |
| 12,13-EPOME | 295.2 | 183 | 50 | -65 | -26 | -9 |
| 13-HODE | 295.2 | 195 | 50 | -140 | -30 | -10 |
| 13-HODE | 295.2 | 113 | 50 | -140 | -30 | -10 |
| 13-HODE | 295.2 | 277 | 50 | -140 | -30 | -10 |
| 9(10)-EpOME | 295.2 | 171.2 | 50 | -60 | -21 | -13 |
| 9,10-EpOME | 295.2 | 171.1 | 50 | -100 | -22 | -13 |
| 9,10-EpOME | 295.2 | 125 | 50 | -100 | -22 | -13 |
| 9,10-EpOME | 295.2 | 183 | 50 | -100 | -22 | -13 |
| 9-HODE | 295.2 | 170.9 | 50 | -130 | -24 | -17 |
| 9-HODE | 295.2 | 207 | 50 | -130 | -24 | -17 |
| 9-HODE | 295.2 | 277 | 50 | -130 | -24 | -17 |
| EPA | 301 | 257 | 50 | -40 | -16 | -24 |
| AA | 303 | 259 | 50 | -60 | -19 | -24 |
| 11-K-12-E-LA 1 (3r) | 309.1 | 181.2 | 50 | -80 | -28 | -9 |
| 11-K-12-E-LA 2 (3 s) | 309.1 | 181.2 | 50 | -150 | -21 | -9 |
| 11-K-12-E-LA 2 (3 s) | 309.1 | 151 | 50 | -150 | -21 | -9 |
| 11-K-12-E-LA 2 (3 s) | 309.1 | 209 | 50 | -150 | -21 | -9 |
| 11-K-12-E-LA 3 (3 t) | 309.1 | 181.2 | 50 | -140 | -22 | -9 |
| 11-K-9-E-LA 1 (3n) | 309.1 | 185.1 | 50 | -60 | -22 | -9 |
| 11-K-9-E-LA2 (3o) | 309.1 | 185.1 | 50 | -140 | -22 | -9 |
| 11-K-9-E-LA2 (3o) | 309.1 | 155 | 50 | -140 | -22 | -9 |
| 11-K-9-E-LA2 (3o) | 309.1 | 171 | 50 | -140 | -22 | -9 |
| 11-K-9-E-LA3 (3p) | 309.1 | 185.1 | 50 | -150 | -22 | -9 |
| 13-K-9-E-LA 1 (3 l) | 309.1 | 171.1 | 50 | -150 | -22 | -15 |
| 13-K-9-E-LA 2 (3 m) | 309.1 | 171.1 | 50 | -150 | -22 | -15 |
| 13-K-9-E-LA 2 (3 m) | 309.1 | 137 | 50 | -150 | -22 | -15 |
| 13-K-9-E-LA 2 (3 m) | 309.1 | 155 | 50 | -150 | -22 | -15 |
| 9-K-12-E-LA | 309.1 | 209.2 | 50 | -70 | -26 | -15 |
| 9-K-12-E-LA | 309.1 | 165 | 50 | -70 | -26 | -15 |
| 9-K-12-E-LA | 309.1 | 193 | 50 | -70 | -26 | -15 |
| 11-H-12-E-LA 1 (3 g) | 311.2 | 197.1 | 50 | -80 | -24 | -15 |
| 11-H-12-E-LA 2 (3 h) | 311.2 | 197.1 | 50 | -90 | -26 | -15 |
| 11-H-12-E-LA 2 (3 h) | 311.2 | 181 | 50 | -90 | -26 | -15 |
| 11-H-12-E-LA 2 (3 h) | 311.2 | 211 | 50 | -90 | -26 | -15 |
| 11-H-9-E-LA 1 (3c) | 311.2 | 201.1 | 50 | -90 | -24 | -29 |
| 11-H-9-E-LA2 (3d) | 311.2 | 201.1 | 50 | -90 | -24 | -29 |
| 11-H-9-E-LA2 (3d) | 311.2 | 171 | 50 | -90 | -24 | -29 |
| 11-H-9-E-LA2 (3d) | 311.2 | 185 | 50 | -90 | -24 | -29 |
| 13-H-9-E-LA | 311.2 | 171 | 50 | -90 | -28 | -15 |
| 13-H-9-E-LA | 311.2 | 139 | 50 | -90 | -28 | -15 |
| 13-H-9-E-LA | 311.2 | 211 | 50 | -90 | -28 | -15 |
| 9-H-12-E-LA | 311.2 | 193 | 50 | -110 | -24 | -13 |
| 9-H-12-E-LA | 311.2 | 171 | 50 | -110 | -24 | -13 |
| 9-H-12-E-LA | 311.2 | 211 | 50 | -110 | -24 | -13 |
| 9-HpODE | 311.2 | 185.2 | 50 | -60 | -22 | -16 |
| 12,13-DiHOME | 313.2 | 183.1 | 50 | -115 | -28 | -13 |
| 12,13-DiHOME | 313.2 | 129 | 50 | -115 | -28 | -13 |
| 12,13-DiHOME | 313.2 | 195 | 50 | -115 | -28 | -13 |
| 9,10-DiHOME | 313.2 | 201.1 | 50 | -140 | -28 | -17 |
| 9,10-DiHOME | 313.2 | 125 | 50 | -140 | -28 | -17 |
| 9,10-DiHOME | 313.2 | 171 | 50 | -140 | -28 | -17 |
| 15-d-PGA2 | 315 | 255 | 50 | -40 | -20 | -17 |
| 15-deoxy-PGJ2 | 315 | 271 | 50 | −60 | −18 | −24 |
| 15-deoxy-PGJ2 | 315.2 | 203 | 50 | -30 | -20 | -17 |
| 11-HEPE | 317 | 215 | 50 | -60 | -20 | -18 |
| 11-HEPE | 317 | 167 | 50 | -60 | -20 | -18 |
| 15-oxo-ETE | 317 | 113 | 50 | -70 | -23 | -11 |
| 8-HEPE | 317 | 155 | 50 | -60 | -18 | -15 |
| 9-HEPE | 317 | 149 | 50 | -60 | -19 | -20 |
| 17,18-EEQ | 317.1 | 215.2 | 50 | -90 | -14 | -17 |
| 17,18-EEQ | 317.1 | 201 | 50 | -90 | -14 | -17 |
| 17,18-EEQ | 317.1 | 259 | 50 | -90 | -14 | -17 |
| 12-HEPE | 317.2 | 179.1 | 50 | -60 | -19 | -18 |
| 12-KETE | 317.2 | 273.3 | 50 | -50 | -23 | -17 |
| 12-KETE | 317.2 | 153 | 50 | -50 | -18 | -17 |
| 14(15)-EpETE (14,15-HEPETE) | 317.2 | 207.1 | 50 | -30 | -19 | -13 |
| 14,15-EEQ | 317.2 | 207 | 50 | -75 | -18 | -19 |
| 14,15-EEQ | 317.2 | 163 | 50 | -75 | -18 | -19 |
| 14,15-EEQ | 317.2 | 175 | 50 | -75 | -18 | -19 |
| 15-HEPE | 317.2 | 219.2 | 50 | -60 | -17 | -22 |
| 15-KETE | 317.2 | 113.2 | 50 | -20 | -18 | -17 |
| 17(18)-EpETE (17,18-HEPETE) | 317.2 | 259.2 | 50 | -40 | -18 | -21 |
| 18-HEPE | 317.2 | 259.1 | 50 | -70 | -16 | -21 |
| 18-HEPE | 317.2 | 161 | 50 | -70 | -16 | -21 |
| 18-HEPE | 317.2 | 215 | 50 | -70 | -16 | -21 |
| 5-HEPE | 317.2 | 115.1 | 50 | -75 | -18 | -9 |
| 5-KETE | 317.2 | 203.2 | 50 | -80 | -25 | -10 |
| 5-oxoETE | 317.2 | 203.2 | 50 | -90 | -24 | -15 |
| 5-oxoETE | 317.2 | 163 | 50 | -90 | -24 | -15 |
| 5-oxoETE | 317.2 | 175 | 50 | -90 | -24 | -15 |
| 5-oxo-ETE | 317.2 | 129 | 50 | -58 | -22 | -18 |
| 16-HETE | 319 | 189 | 50 | -60 | -19 | -21 |
| 16-HETE | 319 | 233 | 50 | -60 | -19 | -21 |
| 17-HETE | 319 | 247 | 50 | -60 | -22 | -21 |
| 18-HETE | 319 | 261 | 50 | -60 | -23 | -18 |
| 19-HETE | 319 | 231 | 50 | -58 | -21 | -10 |
| 5-HETE | 319 | 115 | 50 | −70 | −19 | −15 |
| 11-HETE | 319.1 | 167.2 | 50 | -65 | -22 | -11 |
| 11-HETE | 319.1 | 149 | 50 | -65 | -22 | -11 |
| 11-HETE | 319.1 | 195 | 50 | -65 | -22 | -11 |
| 11(12)-EpETrE | 319.2 | 167.1 | 50 | -30 | -17 | -12 |
| 11,12-EET | 319.2 | 208 | 50 | -105 | -20 | -15 |
| 11,12-EET | 319.2 | 167 | 50 | -105 | -20 | -15 |
| 11,12-EET | 319.2 | 179 | 50 | -105 | -20 | -15 |
| 11-HETE | 319.2 | 301 | 50 | -65 | -22 | -11 |
| 12-HETE | 319.2 | 135 | 50 | -85 | -20 | -17 |
| 12-HETE | 319.2 | 163 | 50 | -85 | -20 | -17 |
| 12-HETE | 319.2 | 179 | 50 | -85 | -20 | -17 |
| 14(15)-EpETrE | 319.2 | 175 | 50 | -30 | -17 | -13 |
| 14(15)-EpETrE | 319.2 | 175 | 50 | -30 | -17 | -13 |
| 14,15-EET | 319.2 | 219.1 | 50 | -90 | -16 | -15 |
| 14,15-EET | 319.2 | 113 | 50 | -90 | -16 | -15 |
| 14,15-EET | 319.2 | 175 | 50 | -90 | -16 | -15 |
| 15-HETE | 319.2 | 219.1 | 50 | -75 | -18 | -17 |
| 15-HETE | 319.2 | 175 | 50 | -75 | -18 | -17 |
| 15-HETE | 319.2 | 203 | 50 | -75 | -18 | -17 |
| 15-HETE | 319.2 | 301.4 | 50 | -75 | -18 | -17 |
| 20-HETE | 319.2 | 245.1 | 50 | -115 | -20 | -23 |
| 20-HETE | 319.2 | 163 | 50 | -115 | -20 | -23 |
| 20-HETE | 319.2 | 289 | 50 | -115 | -20 | -23 |
| 20-HETE | 319.2 | 275.2 | 50 | -75 | -25 | -22 |
| 5(6)-EpETrE | 319.2 | 191.2 | 50 | -30 | -17 | -10 |
| 5,6-EET | 319.2 | 191.2 | 50 | -25 | -16 | -13 |
| 5,6-EET | 319.2 | 115 | 50 | -25 | -16 | -13 |
| 5,6-EET | 319.2 | 163 | 50 | -25 | -16 | -13 |
| 5-HETE | 319.2 | 115 | 50 | -75 | -18 | -9 |
| 5-HETE | 319.2 | 161 | 50 | -75 | -18 | -9 |
| 5-HETE | 319.2 | 203 | 50 | -75 | -18 | -9 |
| 8(9)-EpETrE | 319.2 | 155.1 | 50 | -50 | -18 | -15 |
| 8.9-EET | 319.2 | 155 | 50 | -95 | -16 | -11 |
| 8.9-EET | 319.2 | 123 | 50 | -95 | -16 | -11 |
| 8.9-EET | 319.2 | 179 | 50 | -95 | -16 | -11 |
| 8-HETE | 319.2 | 155 | 50 | -80 | -20 | -11 |
| 8-HETE | 319.2 | 163 | 50 | -80 | -20 | -11 |
| 8-HETE | 319.2 | 257 | 50 | -80 | -20 | -11 |
| 8-HETE | 319.2 | 301.2 | 50 | -80 | -20 | -11 |
| 9-HETE | 319.2 | 123 | 50 | -85 | -22 | -11 |
| 9-HETE | 319.2 | 167 | 50 | -85 | -22 | -11 |
| 9-HETE | 319.2 | 179 | 50 | -85 | -22 | -11 |
| 9-HETE | 319.2 | 151 | 50 | -70 | -21 | -14 |
| 15-HODE | 321 | 223 | 50 | -30 | -30 | -17 |
| 8-HETRE | 321 | 157 | 50 | -80 | -22 | -11 |
| 15-HETrE | 321.2 | 221.2 | 50 | -30 | -21 | -17 |
| 2,3-dinor-11β-PGF2α | 325.2 | 145.1 | 50 | -30 | -22 | -10 |
| 2,3-dinor-11β-PGF2α | 325.2 | 227 | 50 | -30 | -22 | -10 |
| DHA | 327 | 283 | 50 | -60 | -19 | -24 |
| DHA | 327 | 229 | 50 | -60 | -19 | -24 |
| 9,10,11-TriHOME | 329.2 | 201.1 | 50 | -50 | -30 | -11 |
| 9,10,11-TriHOME | 329.2 | 139 | 50 | -50 | -30 | -11 |
| 9,10,11-TriHOME | 329.2 | 171 | 50 | -50 | -30 | -11 |
| 9,10,13-TriHOME | 329.2 | 171 | 50 | -100 | -30 | -13 |
| 9,10,13-TriHOME | 329.2 | 127 | 50 | -100 | -30 | -13 |
| 9,10,13-TriHOME | 329.2 | 139 | 50 | -100 | -30 | -13 |
| 9,12,13-TriHOME | 329.2 | 211.1 | 50 | -90 | -30 | -11 |
| 9,12,13-TriHOME | 329.2 | 119 | 50 | -90 | -30 | -11 |
| 9,12,13-TriHOME | 329.2 | 229 | 50 | -90 | -30 | -11 |
| 14,15-EET-d11 | 330.3 | 219.2 | 50 | -100 | -15 | -15 |
| ADRINIC | 331 | 287 | 50 | -60 | -19 | -24 |
| PGB3 | 331 | 269 | 50 | -60 | -19 | -19 |
| 20-COOH-AA | 333 | 271 | 50 | -40 | -23 | -10 |
| PGB2 | 333 | 271 | 50 | -15 | -24 | -15 |
| 15-deoxy-PGD2 | 333.2 | 271.2 | 50 | -30 | -22 | -17 |
| bicyclo-PGE2 | 333.2 | 113.2 | 50 | -40 | -30 | -24 |
| bicyclo-PGE2 | 333.2 | 175 | 50 | -40 | -30 | -24 |
| PGA2 | 333.2 | 271.2 | 50 | -15 | -24 | -15 |
| PGB2 | 333.2 | 235 | 50 | -52 | -27 | -10 |
| PGJ2 | 333.2 | 233.1 | 50 | -40 | -22 | -13 |
| PGJ2 | 333.2 | 189 | 50 | -40 | -22 | -13 |
| 14,15-diHETE | 335 | 207 | 50 | -60 | -23 | -19 |
| 5,6-diHETE | 335 | 145 | 50 | -60 | -25 | -13 |
| HXB3 | 335 | 183 | 50 | -40 | -21 | -24 |
| 15-HpETE | 335.2 | 113 | 50 | -30 | -24 | -17 |
| 17,18-DiHETE | 335.2 | 247.2 | 50 | -60 | -22 | -24 |
| 5,15-DiHETE | 335.2 | 115.2 | 50 | -40 | -26 | -10 |
| 5,15-DiHETE | 335.2 | 201 | 50 | -40 | -26 | -10 |
| 5,6-DiHETE (5,5-DIEPE) | 335.2 | 115.1 | 50 | -50 | -29 | -13 |
| 8,15-DiHETE | 335.2 | 235.2 | 50 | -60 | -26 | -15 |
| HXA3 | 335.2 | 273.2 | 50 | -40 | -16 | -24 |
| HXA3 | 335.2 | 195 | 50 | -40 | -16 | -24 |
| LTB4 | 335.2 | 194.9 | 50 | -60 | -22 | -15 |
| LTB4 | 335.2 | 123 | 50 | -60 | -22 | -15 |
| LTB4 | 335.2 | 151 | 50 | -60 | -22 | -15 |
| 14,15-DHET | 337 | 207 | 50 | -60 | -24 | -10 |
| 5,6-DHET | 337 | 145 | 50 | -60 | -26 | -10 |
| 11,12-DiHETrE | 337.2 | 167.2 | 50 | -60 | -28 | -10 |
| 5,6-DiHETrE | 337.2 | 145.1 | 50 | -40 | -22 | -13 |
| 8,9-DiHETrE | 337.2 | 127.1 | 50 | -60 | -30 | -10 |
| 10-HDoHE | 343 | 153 | 50 | -60 | -21 | -12 |
| 11-HDOHE | 343 | 149 | 50 | -60 | -19 | -10 |
| 11-HDoHE | 343 | 121 | 50 | -60 | -19 | -10 |
| 12-HDOHE | 343 | 153 | 50 | -60 | -19 | -10 |
| 13-HDoHE | 343 | 221 | 50 | -60 | -19 | -18 |
| 13-HDoHE | 343 | 193 | 50 | -60 | -19 | -18 |
| 14-HDoHE | 343 | 205 | 50 | -60 | -19 | -14 |
| 14KDPA | 343 | 247 | 50 | -30 | -23 | -17 |
| 16-HDoHE | 343 | 233 | 50 | -60 | -19 | -20 |
| 17-HDoHE | 343 | 245 | 50 | -60 | -18 | -18 |
| 17-KDPA | 343 | 247 | 50 | -60 | -22 | -21 |
| 19,20-EDP | 343 | 299 | 50 | -60 | -16 | -24 |
| 20-HDoHE | 343 | 241 | 50 | -60 | -19 | -22 |
| 4-HDoHE | 343 | 101 | 50 | -60 | -21 | -10 |
| 8-HDoHE | 343 | 109 | 50 | -60 | -19 | -11 |
| 7-HDoHE | 343 | 141 | 50 | -60 | -19 | -12 |
| 10-HDHA | 343.2 | 153.1 | 50 | -95 | -22 | -17 |
| 10-HDHA | 343.2 | 161 | 50 | -95 | -22 | -17 |
| 10-HDHA | 343.2 | 261 | 50 | -95 | -22 | -17 |
| 14-HDHA | 343.2 | 205.1 | 50 | -85 | -16 | -17 |
| 14-HDHA | 343.2 | 161 | 50 | -85 | -16 | -17 |
| 14-HDHA | 343.2 | 233 | 50 | -85 | -16 | -17 |
| 16(17)-EpDPE | 343.2 | 233.2 | 50 | -40 | -19 | -11 |
| 16(17)-EpDPE | 343.2 | 193 | 50 | -40 | -19 | -11 |
| 16,17-EDP | 343.2 | 245.2 | 50 | -100 | -15 | -15 |
| 16,17-EDP | 343.2 | 147 | 50 | -100 | -15 | -15 |
| 16,17-EDP | 343.2 | 201 | 50 | -100 | -15 | -15 |
| 16,17-EDP | 343.2 | 274 | 50 | -60 | -15 | -24 |
| 17-HDHA | 343.2 | 245.1 | 50 | -85 | -20 | -15 |
| 17-HDHA | 343.2 | 147 | 50 | -85 | -20 | -15 |
| 17-HDHA | 343.2 | 201 | 50 | -85 | -20 | -15 |
| 17-HDoHE | 343.2 | 281.3 | 50 | -60 | -18 | -18 |
| 17-HDoHE | 343.2 | 229 | 50 | -60 | -18 | -18 |
| 19(20)-EpDPE | 343.2 | 281.3 | 50 | -50 | -18 | -18 |
| 19(20)-EpDPE | 343.2 | 241 | 50 | -50 | -18 | -18 |
| 19,20-EDP | 343.2 | 241.2 | 50 | -115 | -16 | -15 |
| 19,20-EDP | 343.2 | 133 | 50 | -115 | -16 | -15 |
| 19,20-EDP | 343.2 | 187 | 50 | -115 | -16 | -15 |
| 4-HDHA | 343.2 | 101 | 50 | -95 | -18 | -7 |
| 4-HDHA | 343.2 | 133 | 50 | -95 | -18 | -7 |
| 4-HDHA | 343.2 | 241 | 50 | -95 | -18 | -7 |
| 7-HDHA | 343.2 | 141.1 | 50 | -70 | -18 | -11 |
| 7-HDHA | 343.2 | 113 | 50 | -70 | -18 | -11 |
| 7-HDHA | 343.2 | 201 | 50 | -70 | -18 | -11 |
| 15-keto PGD2 | 349 | 235 | 50 | -30 | -40 | -17 |
| 15-keto PGE2 | 349 | 235 | 50 | -30 | -26 | -17 |
| LXA5 | 349 | 215 | 50 | -70 | -20 | -9 |
| PGD3 | 349.2 | 269.2 | 50 | -60 | -19 | -20 |
| PGE3 | 349.2 | 269.2 | 50 | -60 | -20 | -18 |
| PGK2 | 349.2 | 205.1 | 50 | -40 | -31 | -13 |
| PGK2 | 349.2 | 249 | 50 | -40 | -31 | -13 |
| 15R-LXA4 | 351 | 165 | 50 | -70 | -23 | -11 |
| DHK-PGE2 | 351 | 175.2 | 50 | -40 | -26 | -24 |
| DHK-PGE2 | 351 | 207 | 50 | -40 | -26 | -24 |
| PGF3α | 351 | 193 | 50 | -60 | -30 | -16 |
| PGD2 | 351 | 271 | 50 | -60 | -24 | -24 |
| 11β-PGE2 | 351.2 | 271.2 | 50 | -40 | -23 | -9 |
| 15-keto PGF2α | 351.2 | 219.1 | 50 | -50 | -32 | -17 |
| 20-hydroxy LTB4 | 351.2 | 195.1 | 50 | -40 | -23 | -22 |
| DHK-PGD2 | 351.2 | 175.2 | 50 | -40 | -26 | -24 |
| DHK-PGD2 | 351.2 | 207 | 50 | -40 | -26 | -24 |
| LXA4 | 351.2 | 114.9 | 50 | -70 | -20 | -9 |
| LXA4 | 351.2 | 217 | 50 | -70 | -20 | -9 |
| LXA4 | 351.2 | 235 | 50 | -70 | -20 | -9 |
| LXB4 | 351.2 | 221.1 | 50 | -90 | -22 | -25 |
| LXB4 | 351.2 | 189 | 50 | -25 | -25 | -25 |
| LXB4 | 351.2 | 233 | 50 | -25 | -25 | -25 |
| PGE2 | 351.2 | 271.2 | 50 | -15 | -24 | -15 |
| PGE2 | 351.2 | 189 | 50 | -15 | -24 | -15 |
| PGE2 | 351.2 | 217 | 50 | -15 | -24 | -15 |
| PGE2 | 351.2 | 315.1 | 50 | -15 | -24 | -15 |
| 11β-DHK-PGF2α | 353.2 | 113.2 | 50 | -80 | -47 | -9 |
| 11β-DHK-PGF2α | 353.2 | 221 | 50 | -80 | -47 | -9 |
| 11β-PGF2α | 353.2 | 193.2 | 50 | -50 | -35 | -9 |
| 11β-PGF2α | 353.2 | 335 | 50 | -50 | -35 | -9 |
| 15-keto PGF1α | 353.2 | 221.1 | 50 | -50 | -38 | -17 |
| 8-IsoPGF2α | 353.2 | 193 | 50 | -50 | -34 | -13 |
| 8-IsoPGF2α | 353.2 | 165 | 50 | -50 | -34 | -13 |
| 8-IsoPGF2α | 353.2 | 247 | 50 | -50 | -34 | -13 |
| DHK-PGD1 | 353.2 | 209.1 | 50 | -40 | -26 | -24 |
| DHK-PGF2α | 353.2 | 183.1 | 50 | -80 | -28 | -24 |
| DHK-PGF2α | 353.2 | 291 | 50 | -80 | -28 | -24 |
| PGE1 | 353.2 | 273.2 | 50 | -15 | -24 | -15 |
| PGE1 | 353.2 | 235 | 50 | -15 | -24 | -15 |
| PGF2α | 353.2 | 309 | 50 | -60 | -28 | -24 |
| PGF2α | 353.2 | 193.2 | 50 | -50 | -34 | -13 |
| PGF2α | 353.2 | 165 | 50 | -50 | -34 | -13 |
| PGF2α | 353.2 | 247 | 50 | -50 | -34 | -13 |
| PGF2α | 353.2 | 197 | 50 | -50 | -34 | -13 |
| PGD1 | 353.5 | 271.2 | 50 | -15 | -24 | -15 |
| PGD1 | 353.5 | 273.2 | 50 | -15 | -24 | -15 |
| PGD1 | 353.5 | 235 | 50 | -15 | -24 | -15 |
| DHK-PGF1α | 355.2 | 193.2 | 50 | -40 | -26 | -24 |
| DH-PGF2α | 355.2 | 275.2 | 50 | -60 | -29 | -24 |
| DH-PGF2α | 355.2 | 283 | 50 | -60 | -29 | -24 |
| DH-PGF2α | 355.2 | 311 | 50 | -60 | -29 | -24 |
| PGF1α | 355.2 | 293.2 | 50 | -50 | -34 | -13 |
| 10S-PROTECTIND1 | 359 | 123 | 50 | -60 | -21 | -12 |
| PDX (10S,17S-DiHDHA) | 359.1 | 153 | 50 | -90 | -22 | -13 |
| PDX (10S,17S-DiHDHA) | 359.1 | 137 | 50 | -90 | -22 | -13 |
| PDX (10S,17S-DiHDHA) | 359.1 | 261 | 50 | -90 | -22 | -13 |
| MaR1 | 359.2 | 250.1 | 50 | -90 | -20 | -19 |
| MaR1 | 359.2 | 177 | 50 | -90 | -20 | -19 |
| MaR1 | 359.2 | 221 | 50 | -90 | -20 | -19 |
| 10(S),17(S)-DiHDoHE | 359.2 | 153.2 | 50 | -90 | -20 | -19 |
| PD1/NPD1 | 359.2 | 153 | 50 | -115 | -20 | -13 |
| PD1/NPD1 | 359.2 | 137 | 50 | -115 | -20 | -13 |
| PD1/NPD1 | 359.2 | 261 | 50 | -115 | -20 | -13 |
| dihomo PGJ2 | 361 | 299 | 50 | -40 | -37 | -24 |
| 19,20-DiHDPA | 361.2 | 273.3 | 50 | -40 | -22 | -18 |
| 19,20-DiHDPA | 361.2 | 229 | 50 | -40 | -22 | -18 |
| 20-carboxy LTB4 | 365.2 | 303 | 50 | -40 | -26 | -10 |
| 20-carboxy LTB4 | 365.2 | 347.2 | 50 | -40 | -26 | -10 |
| 19-hydroxy PGE2 | 367 | 243 | 50 | -20 | -31 | -10 |
| 20-hydroxy PGE2 | 367 | 175 | 50 | -30 | -27 | -22 |
| 6-keto PGE1 | 367.2 | 143.1 | 50 | -60 | -25 | -15 |
| 6-keto PGE1 | 367.2 | 331 | 50 | -60 | -25 | -15 |
| 20-hydroxy PGF2α | 369.2 | 193.1 | 50 | -40 | -39 | -22 |
| 20-hydroxy PGF2α | 369.2 | 165 | 50 | -40 | -39 | -22 |
| 6-keto-PGF1α | 369.2 | 163.1 | 50 | -60 | -36 | -10 |
| 6-keto-PGF1α | 369.2 | 245 | 50 | -60 | -36 | -10 |
| TXB2 | 369.2 | 169.1 | 50 | -100 | -15 | -15 |
| TXB2 | 369.2 | 177 | 50 | -100 | -15 | -15 |
| TXB2 | 369.2 | 195 | 50 | -100 | -15 | -15 |
| TXB1 | 371.2 | 171.1 | 50 | -100 | -15 | -15 |
| RvD1 | 375.2 | 215.1 | 50 | -70 | -26 | -15 |
| RvD1 | 375.2 | 141 | 50 | -70 | -26 | -15 |
| RvD1 | 375.2 | 233 | 50 | -70 | -26 | -15 |
| RvD2 | 375.2 | 141 | 50 | -70 | -26 | -15 |
| RvD2 | 375.2 | 175 | 50 | -70 | -26 | -15 |
| RvD2 | 375.2 | 215 | 50 | -70 | -26 | -15 |
| RvD3 | 375.3 | 147.1 | 50 | -145 | -26 | -39 |
| RvD3 | 375.3 | 137 | 50 | -145 | -26 | -39 |
| RvD3 | 375.3 | 181 | 50 | -145 | -26 | -39 |
| RvD4 | 375.3 | 101 | 50 | -75 | -28 | -9 |
| RvD4 | 375.3 | 225 | 50 | -75 | -28 | -9 |
| RvD4 | 375.3 | 131 | 50 | -75 | -28 | -9 |
| dihomo PGE2 | 379 | 299 | 50 | -40 | -37 | -24 |
| 1α,1β-dihomo-PGF2α | 381.3 | 337.2 | 50 | -40 | -37 | -10 |
| LTE4 | 438.2 | 333.2 | 50 | -60 | -22 | -15 |
| LTD4 | 495.2 | 177.1 | 50 | -60 | -22 | -15 |
| LTC4 | 624.3 | 272.1 | 50 | -60 | -22 | -15 |
| TXB3 | 367.2 | 169.1 | 50 | -100 | -15 | -15 |


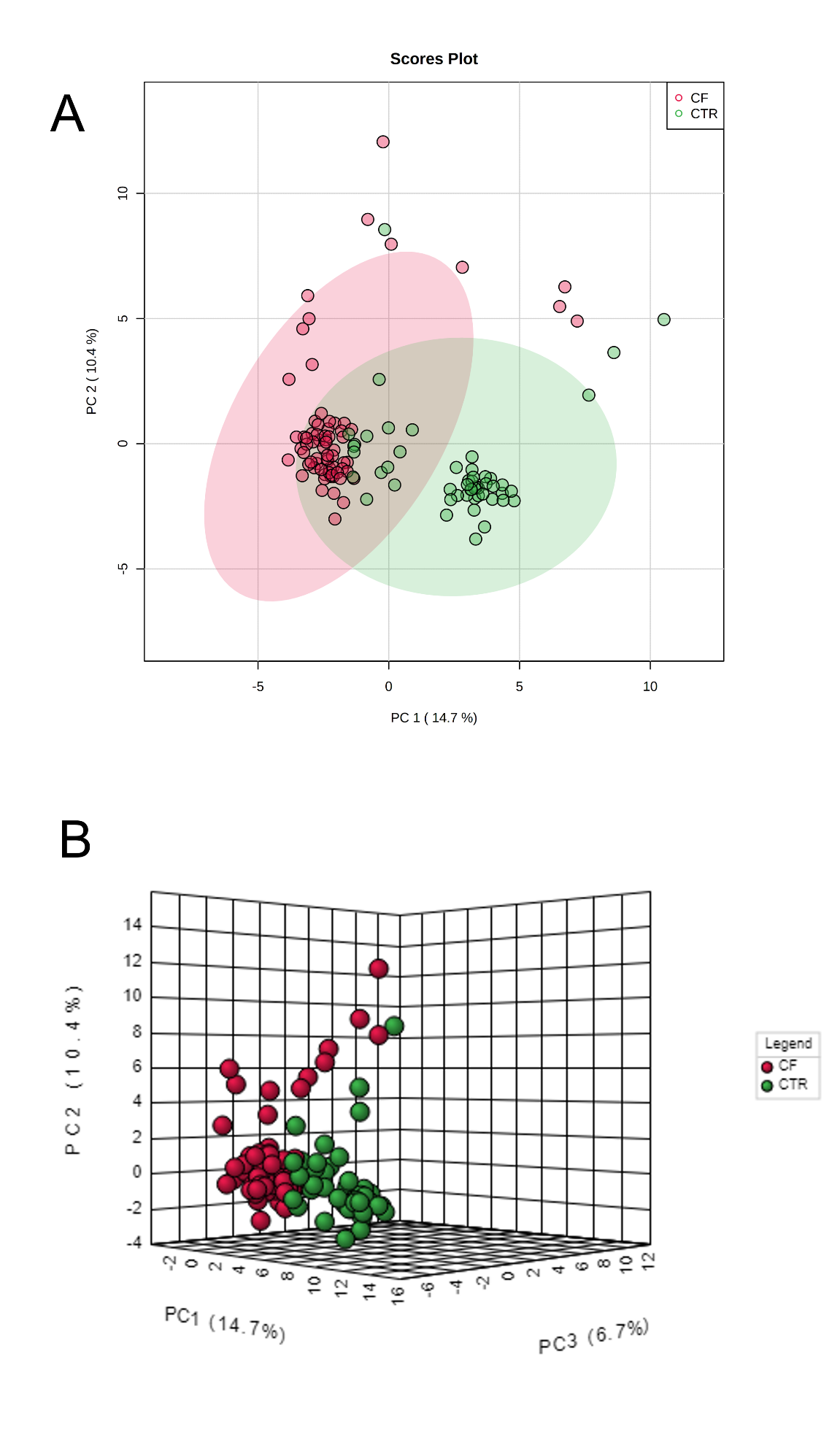


**Supplementary Figure 1**. Principal Component Analysis (PCA) analysis discriminating patients with cystic fibrosis (CF) and controls (CTR). A: 2D score plot; B: 3D score plot. PC: principal component.
